# Ultraviolet Photodetection Based on High-Performance Co-Plus-Ni Doped ZnO Nanorods Grown by Hydrothermal Method on Transparent Plastic Substrate

**DOI:** 10.3390/nano10061225

**Published:** 2020-06-23

**Authors:** Hafiz Muhammad Salman Ajmal, Fasihullah Khan, Kiyun Nam, Hae Young Kim, Sam Dong Kim

**Affiliations:** Division of Electronics and Electrical Engineering, Dongguk University, Seoul 100–715, Korea; salman.gikian@gmail.com (H.M.S.A.); fasihullah.khan@dongguk.edu (F.K.); kynamkr@gmail.com (K.N.); fridayhy@naver.com (H.Y.K.)

**Keywords:** UV detector, codoping in ZnO, ZnO nanorods, plastic substrate, spectral responsivity

## Abstract

A growth scheme at a low processing temperature for high crystalline-quality of ZnO nanostructures can be a prime stepping stone for the future of various optoelectronic devices manufactured on transparent plastic substrates. In this study, ZnO nanorods (NRs) grown by the hydrothermal method at 150 °C through doping of transition metals (TMs), such as Co, Ni, or Co-plus-Ni, on polyethylene terephthalate substrates were investigated by various surface analysis methods. The TM dopants in ZnO NRs suppressed the density of various native defect-states as revealed by our photoluminescence and X-ray photoelectron spectroscopy analysis. Further investigation also showed the doping into ZnO NRs brought about a clear improvement in carrier mobility from 0.81 to 3.95 cm^2^/V-s as well as significant recovery in stoichiometric contents of oxygen. Ultra-violet photodetectors fabricated with Co-plus-Ni codoped NRs grown on an interdigitated electrode structure exhibited a high spectral response of ~137 A/W, on/off current ratio of ~135, and an improvement in transient response speed with rise-up and fall-down times of ~2.2 and ~3.1 s, respectively.

## 1. Introduction

Technological advancements of flexible microelectronics are revolutionizing the world by introducing wearable and implantable sensor devices such as wrist-bands and soft exosuits for the healthcare framework. Such gadgets can precisely measure the blood pressure, heart rate, electrocardiogram, and respiration rate of a human body in real-time [1,2]. Transparent plastic substrates have been highlighted in response to savvy innovation of the aforementioned smart device technology since they can be utilized dynamically for the individual to negate any inconvenience or to protect the fabricated sensor on it from damage while using it on a bendable object [3,4,5]. For instance, sensing patches like a tattoo comprising the multiple sensors have been fabricated on several transparent plastic substrates to secure a conformal touch with the curvy portions of the human physique and, in consequence, to have a steady measurement of the physiological variables. Likewise, the sensing devices on plastic substrates can be integrated into several other configurations of wearable designs such as clothing, contact lenses, rings, badges, and glasses [6].

The harmful exposure of ultraviolet (UV) rays to the human body is considered as one of the vital issues in a wearable system. The UV light falling on the Earth’s surface is about 3–6% of the electromagnetic spectrum (EM) of the sunlight. Approximately 95% of UV EM radiations falling on the globe is UVA ranging in a wavelength range of 315–400 nm, and the remaining portion consists of UVB (280–315 nm), UVC (100–280 nm), and EUV (10–121 nm). The controlled UV irradiance can assist in some health benefits, such as the enhancement of the vitamin-D level in our body and the treatment of skin diseases. However, uncontrolled long exposure to UV can cause various health risks that include irritation in the eyes, skin cancer, retinopathy and premature skin aging [7,8]. The shorter wavelengths EUV and UVC are absorbed almost completely by the ozone layer in the upper atmosphere, and sunblock creams are already available to safeguard against UVB; however, not many solutions were found for the UVA band yet [9]. Therefore, it is essential to monitor the intensity level of UVA on human skin during day-to-day activities by using efficient UV photodetectors (PDs). Fabrication of the UV light sensors synthesized on transparent plastic substrates using various nanostructures has thus become one of the main research interests in recent years. Among various UV compatible nanostructure materials, a wide band gap (3.34 eV), ease of preparation and non-toxicity, chemical and thermal stability of ZnO are substantially favorable properties to fabricate ZnO UV PDs [10].

In order to realize the ZnO nanostructure-based UV sensors on transparent plastic surfaces, a high crystalline quality growth of one-dimensional nanomaterials is obviously one of the critical issues. Recent advances in various nanofabrication schemes have facilitated defect-free growth for the ZnO nanorods (NRs); however, it was revealed by earlier research efforts [11] that NR crystals grown by aqueous routes at low a temperature demonstrate numerous types of defect including the intrinsic donor defects causing the ZnO crystals to behave as n-type. The subject of exact cause for n-type nature in ZnO is still debatable. However, it is believed that the n-type carrier concentration of undoped ZnO crystals can be elicited by the existence of Zn interstitials (Zn_i_) or oxygen vacancies (O_v_) [12]. Another potential cause would be associated with the existence of shallow donor impurities such as the unintentional incorporation of hydrogen during the growth conditions of ZnO nano-crystals [13]. In consequence, a degraded UV sensing efficiency can be observed through these defects due to compromised electron hole pair generation. Furthermore, these defects can induce more increased phonon scattering as well as carrier–carrier scattering which can substantially impact on the performance of UV sensors [14]. Minimizing the native defects and unsolicited donor defect density associated with them by transition metals (TMs) doping, such as Cu [15], Ni [16], or Co [17], is of paramount importance to yield the required optoelectronic properties in ZnO nanocrystals for efficient UV sensing, thereby achieving the high device performance in terms of photoresponse speed, on/off current ratio, and spectral responsivity. Few other reports have proposed polymer coatings or surface functionalization techniques to boost the UV sensor performance.

External impurity incorporation into the ZnO nanocrystals has been intensively investigated as an achievable solution to considerably enhance the performance of UV PDs. For example, Shabannia [18] reported a photoresponse study on highly sensitive Co-doped ZnO NRs based UV PD. Photoresponse study on the doped ZnO thin film by TMs (Mn, Ni, Co) was performed by Rajalakshmi et al. [19]. Dai et al. [20] synthesized p-type Sb-doped and n-type ZnO NRs for PD applications. Beyond the UV sensing purpose, doped ZnO nanostructures have been recently explored by a large number of researchers for water splitting, solar cells, hydrogen generation, and light-emitting diode applications [21,22,23].

In this study, we present UV PDs fabricated on transparent plastic polyethylene terephthalate (PET) substrates using three different dopings in ZnO NRs. The objective of this work is to synthesize the high crystalline-quality ZnO NRs doped by specific TMs, such as Co, Ni, or Co-plus-Ni via a growth solution route under their solubility limits and to compare their sensing performance with that of PDs of the undoped ZnO NRs. Ni and Co dopants were selected as dopants for the synthesis of ZnO NRs because:

(1) Ions of Ni or Co can swap the Zn ions in ZnO nanocrystals without creating any cationic vacancy;
(2) In the host lattice of ZnO, they tend to occupy tetrahedral sites in order to produce the desired physical properties.

In this work, we investigated growth morphologies, optical properties, chemical states on the NR surface, and structural features of the NRs. Performance parameters of the PD devices fabricated with interdigitated electrode (IDE) patterns were assessed in order to substantiate our material characterization for the doped ZnO crystals.

## 2. Experimental Procedure

**Figure 1:**
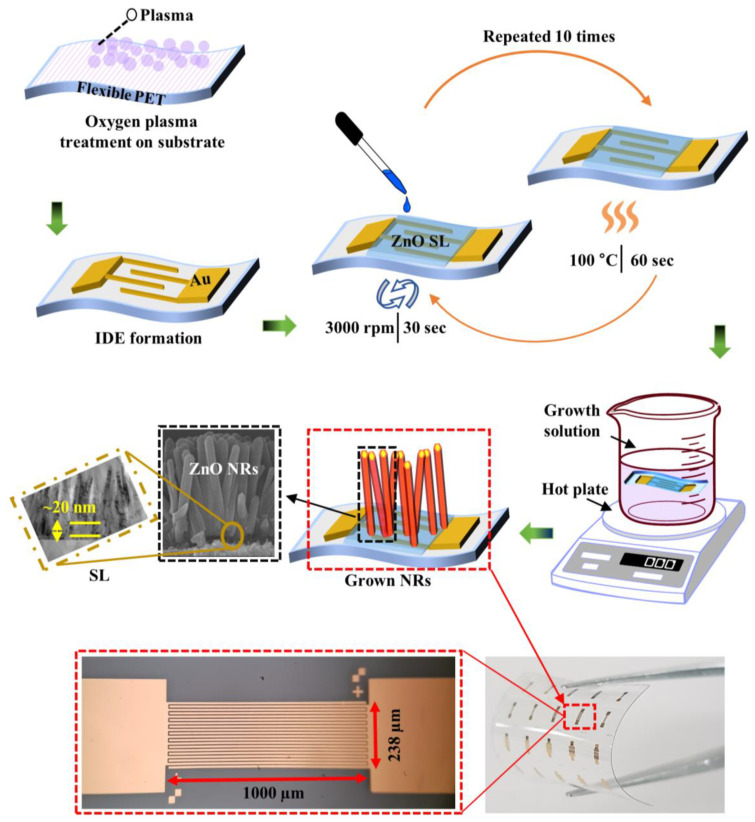
(Top) Schematics of process flow for the ultraviolet (UV) photodetector (PD) devices prepared with ZnO nanorods (NRs) via hydrothermal growth. Cross-sectional scanning electron microscopy (SEM) and transmission electron microscopy (TEM) views of as-grown NRs are shown in the bottom left insets. (Bottom-left) Fabricated PDs on polyethylene terephthalates (PET) substrates and (Bottom-right) a top-view of interdigitated electrode (IDE) pattern.

**Figure 2:**
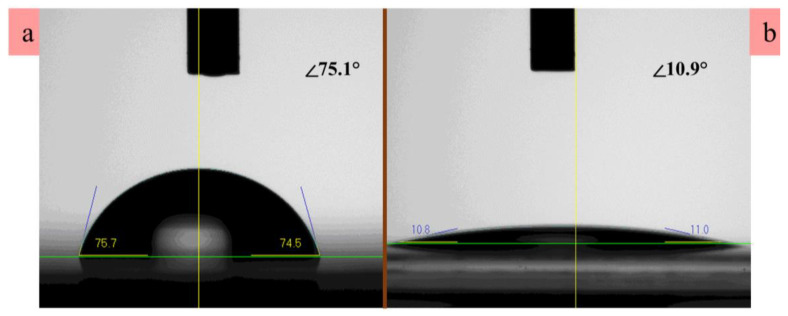
The contact angle measured (**a**) before and (**b**) after oxygen plasma treatment on the surface of the PET substrate.

To synthesise the IDE-patterned UV PDs on transparent plastic substrates, we used commercially available PET substrate of a 2 × 2 cm^2^ dimension. Shown in the top schematics of Figure 1 is a brief illustration of the adopted device process. The chemical reagents (99% as analytically graded) procured from Sigma-Aldrich were used in this study without additional refinement. First, the substrates were washed by deionized (DI) water in a beaker and sonicated in isopropyl alcohol and ethanol sequentially, and finally rinsed by DI water to remove the contaminants. The substrates were then purged by nitrogen gas followed by baking on a hot plate for 1–2 min at 100 °C to completely remove the remaining moisture. The device fabrication process started with patterning of the IDEs by evaporating Au/Ti metal stacks on the substrates and the subsequent growth of ZnO seed-layer (SL) by spin coating. To obtain a hydrophilicity of the PET surface for a good adhesion of Au/Ti metals onto the substrates, we pretreated the substrates in an oxygen (O_2_) plasma condition by using a reactive ion etcher system. The contact angle (CA) was ~75.1° before plasma treatment, and it became significantly smaller (~10.9°) after plasma treatment on the PET surface as illustrated in Figure 2. This result indicates that O_2_ plasma can easily transform the surface state from hydrophobicity to hydrophilicity for the promoted adhesion of SLs and metal electrodes on the PET surface. CA measurements were done by a tensiometer (model No. 250-U4) with an optical goniometer using the sessile drop method. In an ion etcher system, O_2_ exposure was maintained for 5 min with a flow rate of 100 sccm at a RF power of 100 W and a chamber pressure of 50 mTorr. In the next step, we loaded the substrates into a mask aligner (Karl Suss, MA6, 365 nm) to perform photolithography patterning of the IDE structure by using an image-reversal photoresist technique. Subsequently, a metal lift-off process was done by sonication in acetone to acquire the metal IDE patterns after the deposition of 80/30 nm Au/Ti in an electron-beam metal evaporator.

An aqueous solution method was used for the NR synthesis on the IDE patterns. For the SL deposition, a 20 mM concentrated colloidal solution of zinc acetate dehydrate [Zn(CH_3_COO)_2_·2H_2_O] in n-propyl alcohol [C_3_H_8_O] was prepared and placed at room temperature for 10–12 h to attain a homogeneous solution. The SL solution was then spun at 3000 rpm for 30 s on the patterned substrates and was annealed on a hotplate at 100 °C for 60 s. This spin-coating was repeated nine to 10 times to produce a thickness of SL~20 nm after annealing as shown by cross-sectional transmission electron microscopy (TEM) in a left inset of Figure 1. The SL deposited samples were post-annealed at ~150 °C for 60 min to evaporate organic residuals and to produce stable crystallites of ZnO. Finally, the growth of ZnO NRs was performed in the following way. In the case of undoped growth solution, an equimolar (25 mM) homogeneous solution of zinc nitrate hexahydrate [Zn(NO_3_)_2_·6H_2_O] and methenamine [C_6_H_12_N_4_] in a glass vessel comprising 200 mL water was kept for stirring at least 1 h to ensure complete mixing. The IDE-patterned substrates were then completely immersed upside down in the growth solution at an elevated temperature of 90 °C for 7–8 h. For Co, Ni doped and Co-plus-Ni codoped NRs, we prepared three separate growth solution batches in the beakers containing 25 mM equimolar Zn(NO_3_)_2_·6H_2_O and C_6_H_12_N_4_. To introduce dopants into each growth solution, we added 2 % cobalt (II) nitrate hexahydrate [Co(NO_3_)_2_·6H_2_O] for Co doping, 2% nickel (II) nitrate hexahydrate [Ni(NO_3_)_2_·6H_2_O] for Ni doping, and 2% of each (Co and Ni) solution for Co-plus-Ni codoping, respectively.

Cross-sectional views and surface morphologies of the NRs were examined by field-emission scanning electron microscopy (FE-SEM, 15 kV, Hitachi 4800S). The crystallinity and preferred orientation of the NRs were examined with X-ray diffraction (XRD, Dmax-2500 Rigaku at 0.154 nm). The oxygen stoichiometry in the chemical bonding of NRs for each doping case was investigated by X-ray photoelectron spectroscopy (XPS, Thermo-fisher scientific, NEXSA) spectrometer with an X-ray source gun of 400 µm spot size. To investigate the optical properties of each NR crystal, photoluminescence (PL) spectroscopy (RPM 2000 Accent, laser excitation at 266 nm, 15 points/s scan rate) as well as ultraviolet–visible (UV–Vis, model T-60) spectroscopy were carried out at the room temperature. Photoresponse measurements of the fabricated PDs within the frame of current-voltages (*I-V*), transient performance by UV light on/off sequence, and the spectral response were probed in our measurement system.

## 3. Results and Discussion

**Figure 3:**
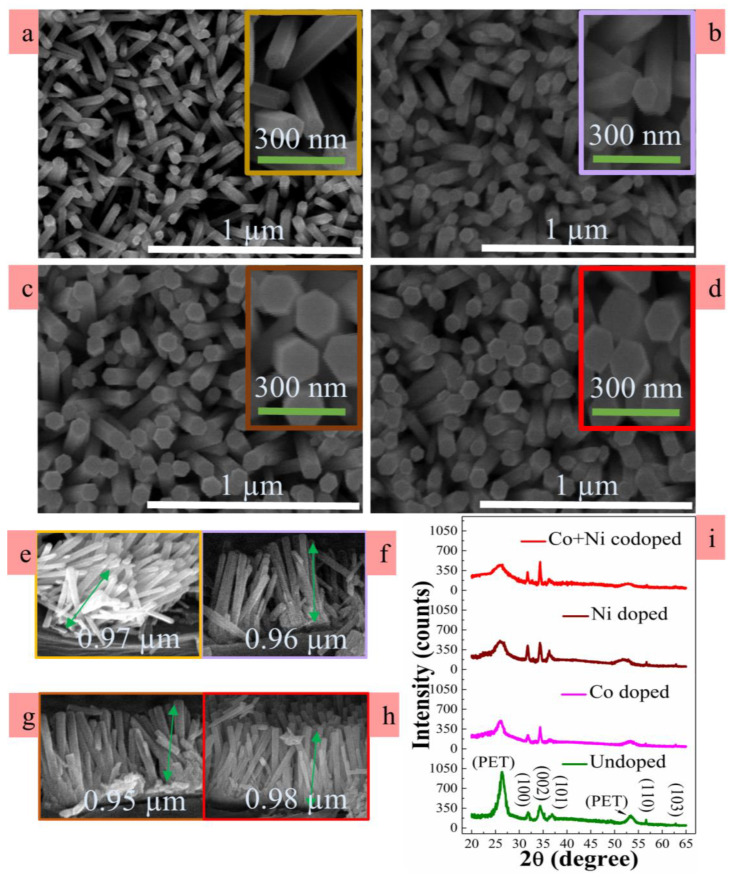
Top views of SEM for (**a**) undoped, (**b**) Co doped, (**c**) Ni doped, and (**d**) Co-plus-Ni codoped ZnO NRs grown on PETs. Cross-sectional SEM views of as-grown ZnO NRs for each case of (**a**–**d**) are also shown in (**e**–**h**). X-ray diffraction (XRD) 2θ patterns of the NRs under different doping conditions are shown in (**i**).

To investigate the effect of different dopings (Co, Ni, and Co-plus-Ni) on the growths of as-grown ZnO NRs, the top views and cross-sectional morphologies were examined using field-emission SEM as depicted in Figure 3a–h. All the NRs exhibited a perfect hexagonal surface of wurtzite crystal structure with flat facets at the top. As shown in top view images, uniform distributions in diameter were obtained from the NRs. In the case of undoped NRs, the measured average diameter was ~73 nm, whereas those in the cases of Co, Ni and Co-plus-Ni dopings were increased to ~109, ~113 and ~119 nm, respectively. Almost the same NR length of ~1 µm on average was observed from each sample of different dopings as shown in Figure 3e–h. The SEM observation indicated the lateral expansion of the NRs was promoted during the growth upon TM doping, and the maximum lateral growth was observed when both Co and Ni dopants were present in NR crystals. This increase in crystal diameter of doped samples can be due to the inhibition of heterogeneous nucleation sites of ZnO. According to classical models for the nucleation, the incorporation of external impurities in ZnO crystals can elevate the energy barrier for the evolution of ZnO crystal nucleus causing the reduced reaction kinetics between OH^-^ and Zn^2+^ in the solution [24,25]. This limited density of ZnO nuclei instead assists lateral expansion of doped ZnO NRs, thereby giving a larger diameter. Total TM concentration in the case of Co-plus-Ni codoping is higher in the growth solution than that of individual TM doping growth solution, and therefore it can lead to a more lateral expansion.

The NRs doped by TMs were grown preferentially in the vertical direction with relatively higher surface density as shown in the SEM views of Figure 3e–h. This result shows a good agreement with the XRD analysis. Two theta (2ϴ) XRD scans were performed from 20° to 65° using a step width of 0.02 degree and a step time of 0.2 s to investigate the crystalline quality and the degree of preferred orientation. As shown in Figure 3i, all the peaks in XRD patterns were matched with the positions of reported standard indexes for Wurtzite ZnO data of JCPDS files (36–1451 card number). The observed diffraction pattern revealed that the hexagonal form of host matrix was fairly well maintained irrespective of the incorporation of Co or Ni. Furthermore, no additional diffraction peaks associated to any phase of Ni, NiO, Co, or CoO or dopant traces except peaks from PET were detected in our XRD resolution limit [26]. This means that Co or Ni dopants did not produce any secondary compound phases during the NR growth but were fairly well dissolved as dopants into the host lattice matrix by replacing Zn sites. The most intense peak was observed from (002) reflection at 2ϴ = 34.5° ± 0.02° from every specimen, which specifies that the intrinsic growth directions of ZnO crystals were well aligned to *c*-axis normal to the substrate. As reported in the earlier research articles [27,28,29], the preferential (002) growth is associated with its lowest surface free energy and the maximum atomic packing density of (002). The reflection intensities from (002) peaks were increased after Co, Ni, doping or Co-plus-Ni codoping, and this indicates the improvement of (002) preferred growth of TM-doped ZnO NRs.

Table 1 outlines important XRD parameters extracted from each NR crystal, such as intensity and position of (002) reflections, (002) full width at half maximum (FWHM), estimated lattice constant *c*, and degree of (002) orientation. The (002) FWHM was greatly reduced with TM dopant incorporation compared to that of undoped sample. This can be related to the increased diameter of NR crystals after doping in pristine ZnO NRs [30]. The (002) reflections from TM-doped NRs were shifted slightly toward higher angles than that of the undoped ones, and this result was in good agreement with our recent Cu doping experimental observation [31], which validates the potential substitution of Zn^2+^ by either Ni^2+^ or Co^2+^ in the host crystal of ZnO. However, a small amount of shift in (002) reflection can be associated with the insignificant mismatch in atomic radii of Ni (0.69 Å) or Co (0.72 Å) with that of Zn (0.74 Å) [32,33].

The modified lattice parameter *c* caused by the replacement of TM dopants was calculated in the following expression [34]:

(1) $$\frac{1}{d{\left(hkl\right)}^{2}}=\frac{4}{3}\left(\frac{h^{2}+hk+k^{2}}{a^{2}}\right)+\frac{l^{2}}{c^{2}}$$

where *d(hkl)* is the interplanar spacing, *h*, *k*, and *l* represent the miller indices. *a* and *c* are the cell lattice parameters of the hexagonal unit cell. Small amount of shrinkage in *c* (~0.1%) was observed by the dopant incorporation due to the swapping of Zn^2+^ ions by either Co^2+^ or Ni^2+^ as their small mismatch in the ionic-radii [35]. The degree of preferred orientation *F(hkl)* can be calculated in the following formula [36]:

(2) $$F\left(hkl\right)=\left(P\left(hkl\right)-P_{0}\left(hkl\right)\right)/(1-P_{0}\left(hkl\right)$$

where P(hkl)=I(hkl)/ΣI(hkl) and $P_{0}\left(hkl\right)=I_{0}\left(hkl\right)/\Sigma I_{0}\left(hkl\right)\cdot I\left(hkl\right)$ and $I_{0}\left(hkl\right)$ indicate the measured peak intensity and reference peak intensity given by JCPDS (36-1451) of (hkl) plane, respectively. ΣI(hkl) and $I_{0}\left(hkl\right)$ represent the summation of measured peak intensities and reference peak intensities, respectively. As shown in Table 1, the increase in *F(002)* after the TM doping supports the promoted *c*-axis alignment of doped NR crystals.

**Figure 4:**
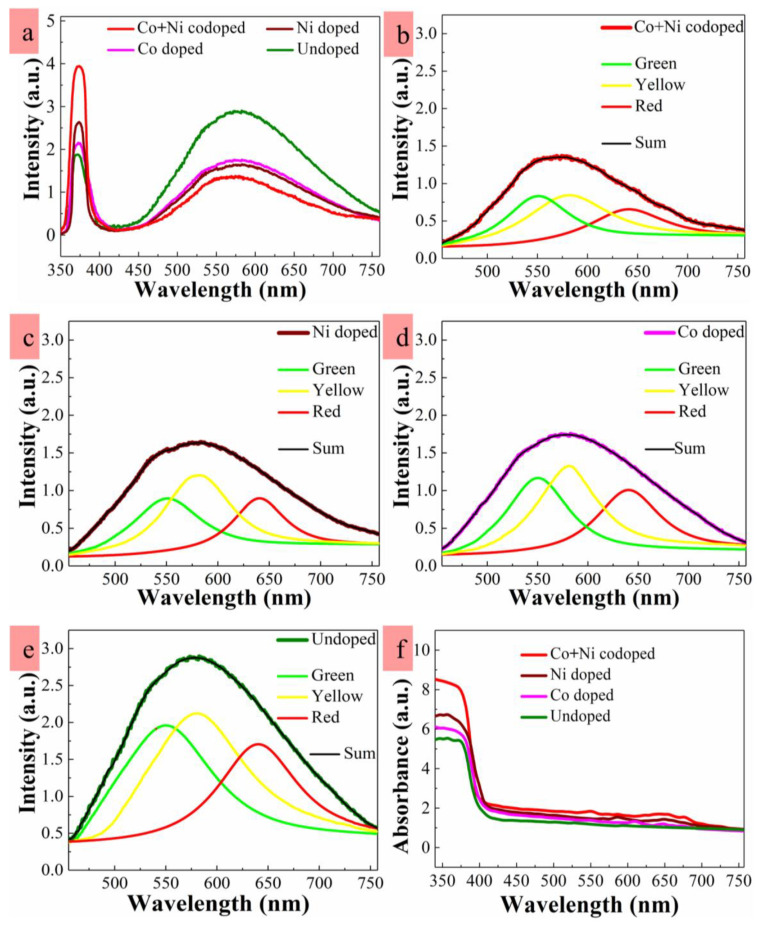
(**a**) Photoluminescence (PL) spectra of the NRs measured at room temperature and deconvoluted visible emission spectra of the NRs grown with (**b**) Co-plus-Ni doping, (**c**) Ni doping, (**d**) Co doping, and (**e**) no doping. Ultraviolet (UV)/visible spectra of the NRs measured at room temperature (**f**).

Intrinsic defects present in ZnO NR crystals such as Zn_i_ or O_v_ can affect the optical properties of ZnO nanocrystals, which have been investigated in more depth through great amount of research efforts using PL analysis than those of many other wide bandgap semiconductors. Nonetheless, optical properties of the ZnO nanocrystals when synthesized at low temperature and codoped by Co and Ni have not been fully understood yet. As shown in PL spectra of Figure 4a, we could observe two clear zones of UV emission (350–400 nm) and broad-band visible emission (400–760 nm) from each sample. The UV emission of near band emission (NBE) arises from excitonic band-to-band recombination in ZnO [15,31]. As shown in PL spectra, significantly enhanced UV emissions were shown from the TM-doped nanocrystals. This is attributed to their improved crystalline quality; especially in the case of Co-plus-Ni codoping, the greatest increase of NBE was observed in the spectra. Different amounts of red shift in NBE peak positions were observed at ~372.8, ~372.9, and ~373.3 nm from the samples doped by Co, Ni, and Co-plus-Ni, respectively, compared to ~371.9 nm of undoped crystal. This change in position of UV emission can be originated from a variety of phenomena, such as surface resonance effect due to change in NR surface to volume ratio, band-gap renormalization, lattice distortion, and electron phonon coupling [25,37]. The slight peak shifts observed in this work are most likely due to the relative expansion in the volume of NR caused by TM dopings [24].

The broadband spectral emissions in visible range of 400–760 nm are known to be associated with deep-level emissions (DLEs) caused by the recombination of photo-excited carriers from various categories of intrinsic defects in the crystals. The exact location of these defects and the theoretical judgment of their nature have been still controversial and did not come to a final conclusion [38,39]; however, it will be unavoidable to have various types of intrinsic defects in the ZnO nano-crystals grown as in this work which were synthesized by a solution method at low temperature. Many different residual organic constituents present in growth solution can be incorporated into the growing NR crystals, and they can lead to deep-level defects. These defects cannot be readily eradicated or avoided due to a low processing temperature. TM-doping in ZnO NR crystals can thus be an effective technique to limit the defect related emissions as claimed by earlier studies [24,40]. As illustrated in Figure 4a, the visible emission intensity associated with deep-level defects was highly suppressed by ~1/2 when doped by Co-plus-Ni compared to the emission of undoped NRs.

A more comprehensive analysis using the Gaussian deconvolution technique was carried out on the visible emissions extracted from PL spectra to investigate the difference in origin of DLE for the samples upon doping condition. As depicted in Figure 4a, the ZnO NRs exhibited three distinctive patterns of DLEs centered at 550 nm (green emission at ~2.25 eV), 580 nm (yellow emission at ~2.14 eV), and 640 nm (red emission at ~1.94 eV). The exact nature of the green emission pattern has been the subject of the most debate depending on crystal synthesis method. Oxygen vacancies have been suggested by many investigations, but zinc vacancies, zinc interstitials, oxygen interstitials, and other extrinsic deep levels including Cu also have been proposed to be the single source of this emission [11,27,41]. The yellow emissions were observed in ZnO NRs grown by a chemical method at low temperature and known to be associated with the presence of hydroxyl group formed in aqueous solution. It was demonstrated that this emission can be replaced by the green or red ones by post-growth annealing [11,41]. The origin of red emissions is also still uncertain in many aspects, but it has been proposed that it is caused by the transitions associated with zinc vacancy complexes. Oxygen and Zn interstitial are also reported as the primary origin of red emissions [27,42,43].

By dopant incorporation of Co, Ni, and Co-plus-Ni, the green emissions were significantly reduced by 35%, 43%, and 61%, respectively, as shown in Figure 4b–e. Therefore, it can be reasonably assumed that many intrinsic defects related to O/Zn vacancies, interstitials, and anti-sites were reduced in TM-doped crystals. Further substantial changes were also obtained in yellow emissions, where the intensity was suppressed by 31%, 37%, or 51% in the cases of Co, Ni, Co-plus-Ni doping, respectively, compared to that of undoped sample. During the hydrothermal crystal growth, the dissociation of H_2_O on the surface of ZnO at the oxygen vacancy sites can lead to the adsorption of OH species. In the case of TM-doped crystals, the number of oxygen vacancy sites can be reduced because of the strong bonding affinity of TM ions with oxygen ions and a prominent reduction in yellow emission was observed. In red emissions, we observed the similar changing trends in pattern intensity according to the doping type.

Figure 4f demonstrates optical absorption spectra measured from the NRs at room temperature by a UV–Visible spectrometer. The UV absorption of doped samples remained considerably higher than the absorption from the undoped one. The enhanced UV absorption after TM-doping can be due to various causes, such as strain induced by doping and size coarsening of the nano-crystals. In this case, the lateral expansion of NR crystals in diameter and the increase in surface density after TM-doping, as shown in Figure 3, can be the prime factors contributing to the enhancement of absorption. The increase of UV absorption can be also associated with larger crystal volume after the dopant incorporation causing the greater photon absorption due to Mie light scattering [31] that is optimized when the scattering materials have a diameter comparable to or greater than the incident light wavelength. Moreover, the doped NR crystals exhibited the minor absorbance humps in a range of 550 and 690 nm indicating the exchange interaction of ZnO free electrons with the localized electrons in 3d energy levels of dopants. The electronic d–d transitions of dopant levels are also induced in this tetrahedral absorbance edge [44,45].

**Figure 5:**
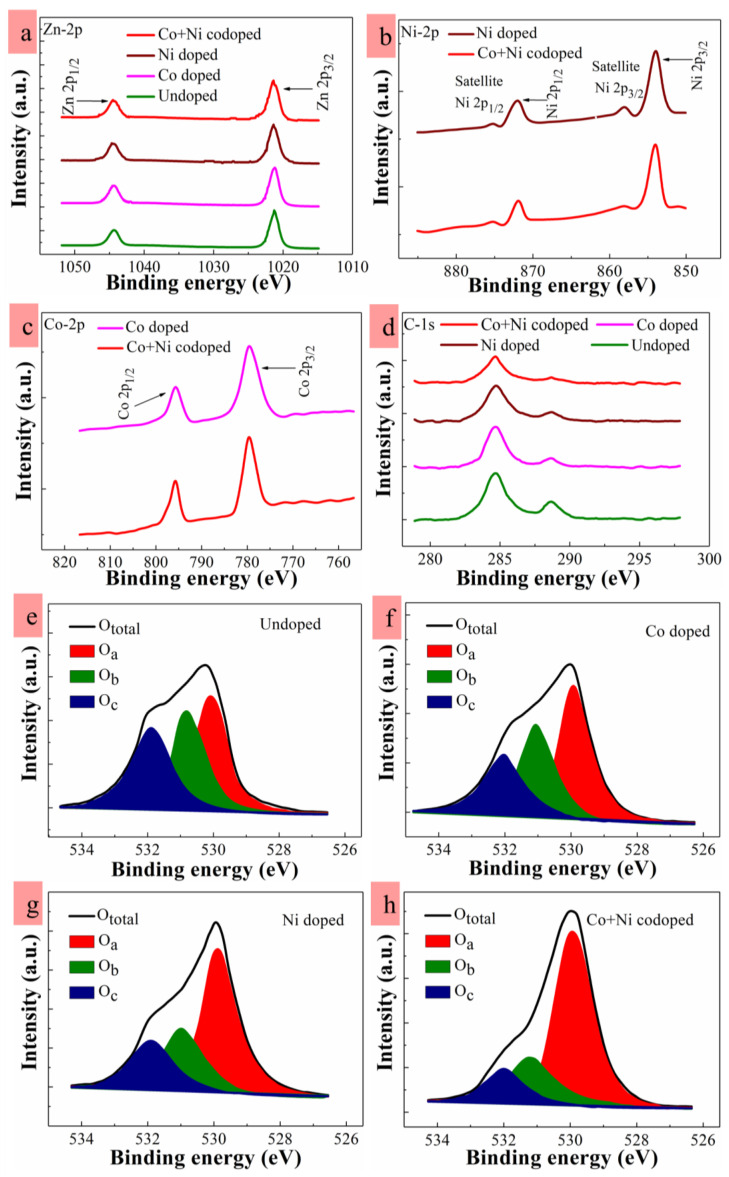
High-resolution X-ray photoelectron spectroscopy (XPS) spectra of (**a**) Zn-2p, (**b**) Ni-2p, (**c**) Co-2p, and (**d**) C-1s of ZnO NRs. Deconvoluted O1s core level spectra into three satellite peaks obtained from (**e**) undoped, (**f**) Co-doped, (**g**) Ni-doped, and (**h**) Co-plus-Ni codoped ZnO NRs.

In order to investigate the bonding states of metal cations (Zn, Co, and Ni) with O anions as well as their atomic concentrations, XPS analysis was carried out in a wide range (0–1200 eV) of binding energies (BE) for the samples. All the relevant elements of zinc, oxygen, carbon and incorporated doping impurities exhibited their respective photoemissions of core-levels and spin-orbital splittings with Auger emissions as revealed in high-resolution spectra. Explicit Zn doublet peaks were highlighted as Zn-2p_3/2_ (~1020.8 ± 0.1 eV) and Zn-2p_1/2_ (~1044.1 ± 0.1 eV) [see Figure 5a]. Symmetrical outlines of the observed photoemission peaks, their positions, and spin orbital splitting level at ~23.3 eV of the Zn-2p doublet substantiate the factual presence of Zn^2+^ chemical state in the stoichiometry of ZnO matrix [39]. Shown in Figure 5b,c are the collected photoemission peaks from Co-2p and Ni-2p core levels, respectively. In the cases of Co and Co-plus-Ni dopings, the Co-2p peak patterns splitting into Co-2p_3/2_ (~779.3 eV) and Co-2p_1/2_ (~795.1 eV) were clearly detected in a specific range of XPS. No peak was observed from ~778.0 or ~793.0 eV corresponding to the metallic traces of Co in bi-cationic state. The resulting energy split between Co-2p_1/2_ and Co-2p_3/2_ was ~15.9 eV, which indicates the bonding of Co^2+^ ions (~15.5 eV required) in the host crystals [44,46]. The Co atomic concentrations estimated from all the samples of different dopings were summarized in Table 2. In the cases of Ni incorporated dopings, a splitting of Ni-2p core level into two clear photoemission peak patterns of Ni-2p_3/2_ and Ni-2p_1/2_ was shown at ~854.1 and ~872.2 eV with additional two minor peaks at 857.9 and 875.3 eV. These Ni peaks recorded only from Ni and Co-plus-Ni doped NRs crystals were very close to that of the Ni^2+^ ions [44,47], and no peak corresponding to metallic Ni was shown. These XPS observations discussed above suggest that any metallic cluster formation from Co or Ni can be ruled out and that most of TM impurities were blended into the ZnO host matrix as dopants. The emissions from C-1s, as shown in Figure 5d, are in general attributed to surface contamination by hydrocarbons, carbon oxides, and carboxyl groups originated from the solution in the growth stage. In the spectra of C-1s, the first peak of C-C was observed at ~284.6 eV, and the second peak from COOH or COOR was measured at ~288.6 eV. Clear reduction in unwanted carbon impurities after TM dopings can be attributed to the reduced formation of carboxyl groups or oxygenated hydrocarbons caused by the bonding of OH^-^ with carbon oxides, as more reduction in carbon impurity was observed after doping in ZnO nanocrystals with the reduction of OH^-^ and the recovery of stoichiometric oxygen [39]. It can be also postulated that the external TM dopant precursors suppressed the incorporation of carbon impurities into the ZnO crystals by changing the reaction kinetics of carbon complex formation in the solution [48].

Photoemission peaks collected from O-1s spectra were analyzed by applying the Gaussian fitting to examine the detailed statistics of oxygen binding contributions in all samples. For this, O-1s scan spectra were deconvoluted into three distinct secondary peaks denoted by O_a_, O_b_, and O_c_ as shown in Figure 5e–h. The relative ratios of integrated peak intensity of the O-1s satellite peaks are listed in Table 2. The first satellite peak of O_a_ (∼529.8 ± 0.1 eV) is known to have originated from the O^2−^ ion bond formation with the metal cations (Zn^2+^, Co^2+^, Ni^2+^). By taking into account that O_a_ is an optimum estimation for the stoichiometric oxygen entity in the ZnO crystals, we could evaluate the degree of stoichiometry for each sample by estimating the contribution of ∫O_a_ in ∫O_total_, where O_total_ = O_a_ + O_b_ + O_c_, and ∫O_total_ is the summation of curve integrations for three O-1s satellite peaks, and ∫O_a_ represents the curve peak integration of O_a_ [15,27,39]. In a different direct way of evaluating stoichiometry, ∫O_a_/∫O_M_ (denoted by O_a_ /(Zn+dopants)) was also estimated, where ∫O_M_ is the summation of peak curve integrations for Zn, Co, and Ni. As shown in Table 2, the highest O_a_ percentage is shown in the case of Co-plus-Ni doping. Despite the fact that synthesized ZnO crystals are oxygen deficient in nature, the estimated ratio of O_a_ /(Zn+dopants) was significantly enhanced upon TM-doping, and the highest figure of ~0.83 was calculated from the Co-plus-Ni doping. This enhancement in O_a_ can be due to the effective binding of TM metals and oxygen due to greater electronegativity (ζ) of Co (ζ =  1.88) and Ni (ζ  = 1.91) than that of Zn (ζ =  1.65). The O^2-^ ions will favorably form the bonds with TM ions in the host lattice, thereby suppressing the formation of oxygen vacancies in the oxygen-cation bonds [24,31,49]. The second component of O-1s was symbolized as O_b_ (∼531 ± 0.2 eV) and is known to arise from O^2-^ ions in the oxygen-deficient regions (where oxygen vacancies are present) of the ZnO matrix. The third component of the O-1s peak at higher BE (∼532 ± 0.09 eV) expressed by O_c_ is reported to be associated with either a chemisorbed oxygen entity or the species of OH^-^ during the fabrication process in the organic solutions. As shown in Table 2, the presence of O_c_-related defects exhibited a minimum percentage in TM-doped NRs, especially in the case of Co-plus-Ni doping. The degree of stoichiometry O_a_/(Zn+dopants) extracted from undoped samples was ~0.55, and this suggests that a lot of oxygen vacancies are present on the surface. These oxygen vacancies behave as active sites which have a strong affinity to combine with other species [50], which can lead to high contents of OH radicals at oxygen vacancy sites on the NR surface due to dissociation of water or ambient hydrogen. Furthermore, the OH species can combine with carbon impurities to give rise to carboxyl groups or other oxygenated hydrocarbons as represented by the C-1s peaks in the XPS spectra.

In order to investigate the influence of TM-doping on unintentional n-type carrier concentration and electron mobility, we performed Hall measurements at room temperature and a magnetic field strength of 560 mT in a Van der Pauw configuration. As shown in Table 3, the background carrier concentrations were decreased, while the electron mobilities were increased after the dopings. It has been known that the unintentional n-type conductivity in ZnO is caused by the presence of oxygen vacancies or zinc interstitials. Recently, it has been also suggested that H can also substitute for O in ZnO and acts as a shallow donor [12]. The reduction of intrinsic carriers can be attributed to healing of these defects as revealed in our XPS analysis. Neutral complexes can be formed due to exchange interaction of partially filled 3d states of Co or Ni with 4s states of Zn_i_ by the doping of these impurities as Sarkar et al. [51] observed that the formation of Zn_i_ was decreased when Cu doped into ZnO. The mobility feature is related to the decrease in intrinsic defect centers of the donor in the crystals as discussed in our PL and XPS analysis. Therefore, the reduced number of intrinsic donor defects by TM doping can contribute to the suppressed scattering effect in the carrier transport of ZnO NRs [52].

To examine the effect of dopings for the ZnO NRs on the performance of PDs, *I-V* characteristics, transient photoresponse, and spectral response were measured from the UV PDs with IDE patterns fabricated on PET substrates as described in the experimental section in detail. A low biasing voltage of 2 V was used for the practical applications of our UV sensors under a low power operation and to prevent any damage to the plastic substrate by overheating even though higher biasing voltage can aid in the generation of electron-hole pairs in ZnO and enhance the on/off current ratio as well as responsivity. However, the recombination probability of photogenerated carriers can be higher at this low voltage than when measured at high biasing condition; therefore, more sensitive characterization could be expected for the dependence of device performance on the intrinsic defects of ZnO. Transient photoresponse performance of IDE-patterned UV PDs was carried out using an input light source of 300 W Xenon lamp (model No. 300XF). The source light of wide band range (200–800 nm wavelength) was filtered into a single wavelength of 350 nm using a monochromator (model No. CS260-USB-1-MC-D, 1/4 m) with the grating of 2400 lines/mm. The active area of patterned IDE structure was then illuminated by the monochromatic light and probed in a probe station connected with a Keithley source measurement unit. UV light on/off for the transient photoresponse was controlled by a programmable shutter logic control (model No. 71445). All the photoresponse measurements were performed in a dark box.

**Figure 6:**
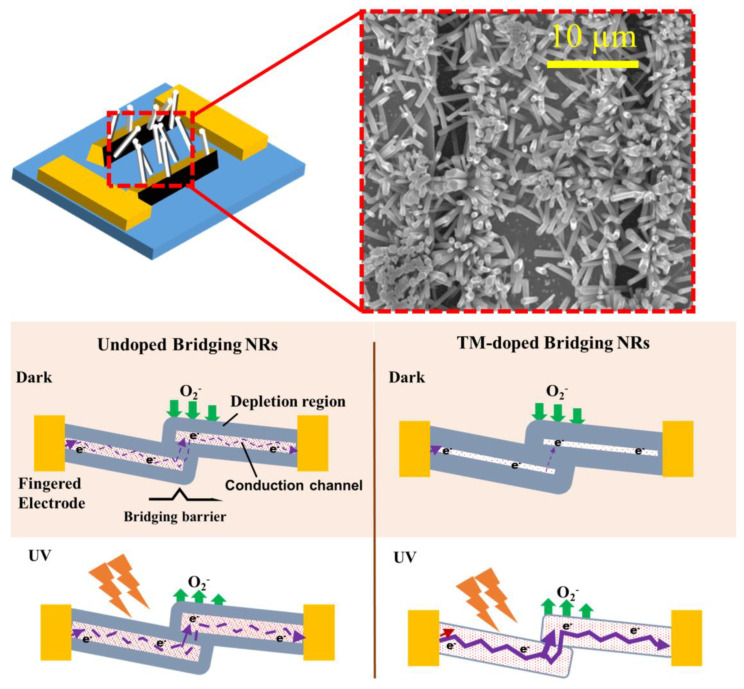
(Top) Schematic illustration of bridging NR structures formed between patterned electrodes and their SEM image shown in the right. (Bottom) Carrier generation and transportation in the ZnO bridging NRs (Left: undoped, Right: transition metal (TM)-doped).

The UV sensing characteristics of our PDs depend on the underlying chemisorption process of ambient oxygen at the ZnO surface [53]. In the dark condition, oxygen molecules on the ZnO surface tend to capture the free electrons in the conduction band level and get adsorbed at the surface by $\left[O_{2}+e^{-}\;\overset{\;}{\to }\;{O_{2}^{-}}_{adsorbed}\right]$, as depicted in Figure 6 (bottom left). Consequently, the surface depletion region at the NR surface is expanded. When the TM-dopants are introduced into ZnO nanocrystals, they can drop the electron concentration *n* (see our Hall measurements in Table 3) due to the reduction of intrinsic defects, such as O_v_ and Zn_i_, as revealed by our PL and XPS analysis and other recent reports [18,54]. 

The depletion layer surface thickness δ of the NR is expressed as follows [55]:

(3) $$\delta =L_{D}{\left(\frac{eV_{s}}{kT}\right)}^{1/2}$$

(4) $$L_{D}={\left(\frac{\varepsilon kT}{e^{2}n}\right)}^{1/2}$$

where *L_D_* is the Debye length, *V_S_* is the adsorbate-induced band bending, *e* is the electronic charge, *kT* is the thermal energy, and *ε* indicates the permittivity. The surface depletion layer of TM-doped ZnO will evolve thicker than that of the undoped one. Since the conduction channels for our PDs comprise the NR–NR junctions, and electrons ought to overcome the bridging energy barrier to maintain the channel conduction from one NR to another as illustrated in Figure 6. On the other hand, the conductance of channel *G* of the bridging NRs is controlled by not only *n* but also dimensional effects, and it can be formulated as [55]:

(5) G=neµπ(D−2δ)/4l

where µ is the electron mobility, *D* is the diameter of the NR, and l represents the length between two electrodes. In the case that the diameter of the synthesized NRs greater than the Debye length as in our study, the prime bottleneck of the charge transport process will occur between the electrodes through the NR-NR junction.

**Figure 7:**
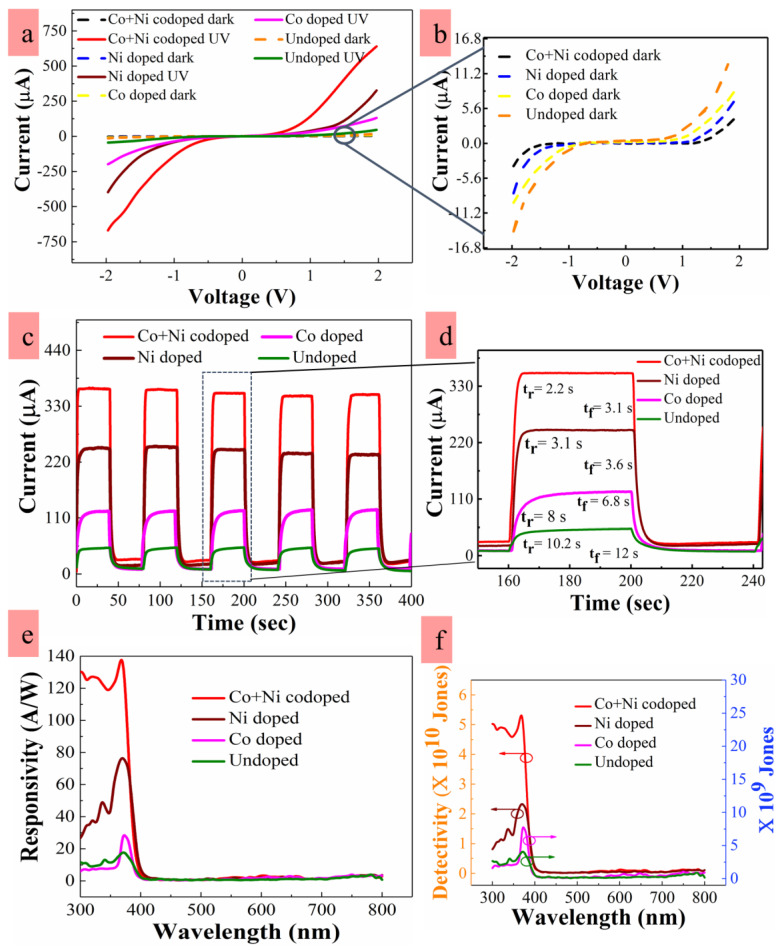
(**a**) Current-voltage (*I*–*V*), (**b**) expanded view of *I*–*V* characteristics in the dark condition, (**c**) transient responses, (**d**) expanded view of single transient cycle, (**e**) spectral responsivities, and (**f**) specific detectivity of PDs.

Figure 7b demonstrates the lowest dark current *I_dark_* of ~4.8 µA of the PDs when doped by Co-plus-Ni. Higher *I_dark_* values of ~8.1, ~10, and ~14 µA were measured from the PDs fabricated with Ni-doped, Co-doped, and undoped NRs, respectively, at a 2 V biasing. The lowest *I_dark_* measured in the case of Co-plus-Ni doping, despite of the largest NR diameter, suggests that charge transport is dominated by the surface depletion region in the NR bridge. UV light illumination generates electron hole pairs, and the generated holes recombine with the electrons captured by ambient oxygen adsorbed on the NR surface [31]. This process drives a desorption phenomenon for the O_2_ molecules (see Figure 7) by [${O_{2}^{-}}_{adsorbed}+h\;\overset{\;hv\;}{\to }O_{2}$]. In consequence, the surface depletion will keep shrinking as long as the generated holes are supplied to the NR surface. UV photocurrent *I_UV_* measured from the PDs fabricated with undoped, Ni-doped, Co-doped, and Co-plus-Ni codoped NRs were ~46, ~330, ~131, and ~649 µA, respectively, as shown in Figure 7a. Carrier trappings in the dark condition to the neutral defect complexes and their detrapping under UV illumination can be responsible for higher *I_UV_* in doped PDs as suggested by Sarkar et al. [51]. The photo-excited carriers quickly recombine with the deep-level defects introduced into the hydrothermally grown ZnO NRs; however, the introduction of TM dopants can significantly suppress the deep-level defects. Therefore, this leads to minimized loss in photogenerated carriers and increases the photocurrent [56].

Improved *I_UV_* in TM-doped PDs can be also attributed to the decrease of bridging barrier thickness as illustrated in Figure 6. The TM dopants incorporated into ZnO can liberate many photogenerated holes from the recombination or trapping to the deep-level defects, thereby shrinking the surface depletion at the NR surface. If the tunneling current is given by an exponential function of bridging barrier thickness, the conduction current through the bridging barrier will be sensitive to any slight change of barrier thickness. Substantial improvement in the UV-to-dark current ratio *(I_UV_/I_dark_)* up to ~135 was achieved from the PD in the case of Co-plus-Ni doping, while a much lower *I_UV_*/*I_dark_* of ~6 was measured from the PD fabricated with undoped NRs.

Transient photoresponse characteristics including the rise-up (response) and fall-down (recovery) times under UV (at 350 nm in this work) on/off conditions is one of important performance parameters for the PDs, where the rise-up and fall-down times represent the time spans of the *I_UV_* reaching up to 90% of its maximum saturation value when UV turns on and of the photocurrent dropping by 90% from its maximum value when UV turns off, respectively. The measured response time t_r_ in the cases of Co-plus-Ni, Ni, and Co doping were ~2.2, ~3.1, and ~8.0 s, respectively. These PDs with doped NRs exhibited much smaller response time than ~10.2 s measured from the PDs with undoped NRs, as shown in Figure 7c,d. The reduction of intrinsic defect density and surface depletion in TM-doped NRs can contribute to the increase in flow rate of photogenerated holes toward the surface of ZnO, thereby accelerating the O_2_ desorption process. The recovery time t_f_ depends upon the O_2_ adsorption process on the ZnO surface which is a strong function of electron diffusional kinetics across the energy barrier built up by surface band bending. The lower surface band bending due to reduced deep-level defect density on the surface can be responsible for the improved recovery time in the cases of TM-doped NRs [39].

One vital figure of merit for the PDs is the spectral responsivity *R* given by [57]:

(6) $$R=\frac{QE\cdot \lambda }{1.24}CF$$

where QE is the quantum efficiency measured at a wavelength of λ, and *CF* is a correction factor given by the dimensions of illuminated beam and device active area. Shown in Figure 7e are the calculated spectral responses of the PDs with an effective active area of 0.238 mm^2^ at 2 V biasing and an optical intensity of 140 µW/cm^2^. The PDs with Co-plus-Ni codoped NRs revealed the highest *R* of ~137 A/W at 370 nm, and this high *R* is due to the enhanced collection of photogenerated electron hole pairs in the form of UV photocurrent with minimum losses by carrier recombination taking place through the defect levels. The increase of *R* can be also related to the enhanced absorption modulated by Mie light scattering mechanism due to the larger sizes of ZnO NRs as discussed earlier. This *R* achieved from the PDs doped by Co-plus-Ni was 10–100 times greater than those measured from the most recent PDs based on ZnO nanostructures fabricated on transparent substrates [58,59].

Specific detectivity D* for a PD is another figure of merit used to characterize performance equal to the reciprocal of noise-equivalent power normalized per square root of the detector area and frequency bandwidth. When the dark current is dominated by the shot noise, D* can be expressed by [60]:

(7) $$D^{\;*}=\sqrt{A}R\;/\sqrt{2qI_{dark}}$$

where *A* is the active area and *q* is the electronic charge. Estimated D* values versus wavelength at 2 V and a light intensity of 140 µW/cm^2^ were illustrated in Figure 7f. The maximum D* of ~5.4 × 10^10^ Jones (cmHz^−1/2^W^−1^) was obtained at 370 nm from the PDs with ZnO NRs doped by Co-plus-Ni. Despite the low process temperature of ~150 °C, the achieved detectivity in this work was comparable to those of the devices with NiZnO (~3.7 × 10^10^ Jones) or MnZnO (~1.6 × 10^10^ Jones) films fabricated at elevated temperatures [61].

## 4. Conclusions

We performed a comparative study to investigate the performance of UV PDs prepared by undoped ZnO NRs grown on IDEs patterns with those of Co, Ni and Co-plus-Ni doped ZnO NRs. The PDs fabricated with Co-plus-Ni codoped NRs showed superior performance in terms of spectral responsivity (~137 A/W), photo-to-dark current as on/off ratio (~135), and transient speed (t_r_ ~2.2 s and t_f_ ~3.1 s) than those of devices with NRs doped by either Co or Ni, and they exhibited much greater differences in term of performance improvement than those of PDs fabricated with undoped NRs. Various surface characterizations, such as PL, XPS, SEM, and XRD, were carried out to investigate the difference in material properties of ZnO nanocrystals that brought about the differences in PD performance. Through these analysis, the incorporation of TM impurities, such as Co or Ni, caused a significant change in the ZnO NR material properties, and in particular, in the case of Co-plus-Ni doping, the most significant improvement was shown in PL properties. The TM doping method at a low temperature processing presented in this work is supposed to be a promising scheme to be used for high-performance PD manufacturing on transparent plastic substrates, and it can be a practical approach for the future research in relevant areas due to its ease of application in wearable devices.

## Figures and Tables

**Table 1:** Parameters of ZnO NR crystals extracted from XRD analysis.

Sample	2*θ* of (002) (Degree)	(002) Intensity (Counts)	FWHM (Degree)	*c* (Å)	Degree of (002) Orientation
Undoped	34.43	385	0.51	5.203	0.22
Co doped	34.45	401	0.37	5.201	0.28
Ni doped	34.45	484	0.35	5.201	0.23
Co + Ni doped	34.47	513	0.30	5.198	0.26

**Table 2:** Atomic percentages of Co, Ni, Zn, and C. Calculated ratios of each O-1s satellite peak and O_a_/(Zn+dopants) for the NR crystals estimated by XPS deconvolution analysis. (at. **%** = atomic percentage).

Sample	Co at.%	Ni at.%	Zn at. %	Oxygen-Peak Deconvolution			O_a_/(Zn + Dopants)	C at.%
				Oa(%)	Ob(%)	Oc (%)		
Undoped	0.00	0.00	37.73	38.31	33.87	27.79	0.55	7.40
Co doped	0.55	0.00	38.52	47.81	30.79	21.98	0.69	4.19
Ni doped	0.00	0.59	38.29	54.55	26.92	19.53	0.79	4.24
Co + Ni doped	0.49	0.37	39.23	58.98	23.73	17.12	0.83	2.86

**Table 3:** Hall measurements of undoped, Co doped, Ni doped, and Co + Ni codoped ZnO NRs.

Sample	Carrier Concentration (n-Type) (cm^−3^)	Electron Mobility (cm^2^/V-s)
Undoped	9.1 × 10^16^	0.81
Co doped	8.5 × 10^15^	1.06
Ni doped	4.4 × 10^15^	1.21
Co + Ni codoped	1.9 × 10^15^	3.95

## References

[1] Guk K., Han G., Lim J., Jeong K., Kang T., Lim E.-K., Jung J. (2019). Evolution of Wearable Devices with Real-Time Disease Monitoring for Personalized Healthcare. Nanomaterials.

[2] Zhang B., Korolj A., Lai B.F.L., Radisic M. (2018). Advances in organ-on-a-chip engineering. Nat. Rev. Mater..

[3] Yeo J.C., Lim C.T. (2016). Emerging flexible and wearable physical sensing platforms for healthcare and biomedical applications. Microsystems Nanoeng..

[4] Palavesam N., Hell W., Drost A., Landesberger C., Kutter C., Bock K. (2020). Influence of Flexibility of the Interconnects on the Dynamic Bending Reliability of Flexible Hybrid Electronics. Electronics.

[5] Ahmad Z., Karimov K.S., Touati F., Ajmal M.S., Ali T., Kayani S.H., Kabutov K., Shakoor R., Al-Thani N. (2016). n-InAs based photo-thermo-electrochemical cells for conversion of solar to electrical energy. J. Electroanal. Chem..

[6] Dang V.Q., Trung T.Q., Duy L.T., Kim B.-Y., Siddiqui S., Lee W., Lee N.-E. (2015). High-Performance Flexible Ultraviolet (UV) Phototransistor Using Hybrid Channel of Vertical ZnO Nanorods and Graphene. ACS Appl. Mater. Interfaces.

[7] Nunez C.G., Vilouras A., Navaraj W.T., Liu F., Dahiya R. (2018). ZnO Nanowires-Based Flexible UV Photodetector System for Wearable Dosimetry. IEEE Sensors J..

[8] Kurz W., Yetisen A.K., Kaito M.V., Fuchter M.J., Jakobi M., Elsner M., Koch A.W. (2020). UV-Sensitive Wearable Devices for Colorimetric Monitoring of UV Exposure. Adv. Opt. Mater..

[9] Nishino K., Haryu Y., Kinoshita A., Nakauchi S. (2019). Development of the multispectral UV polarization reflectance imaging system (MUPRIS) for in situ monitoring of the UV protection efficacy of sunscreen on human skin. Ski. Res. Technol..

[10] Belhaj M., Dridi C., Yatskiv R., Grym J. (2020). The improvement of UV photodetection based on polymer/ZnO nanorod heterojunctions. Org. Electron..

[11] Tam K.H., Cheung C.K., Leung Y.H., Djurišić A.B., Ling C.C., Beling C.D., Fung S., Kwok W.-M., Chan W.K., Phillips D.L. (2006). Defects in ZnO Nanorods Prepared by a Hydrothermal Method. J. Phys. Chem. B.

[12] Janotti A., van de Walle C.G. (2009). Fundamentals of zinc oxide as a semiconductor. Rep. Prog. Phys..

[13] Feng W., Cho S., Wang M.-S., Dung D.D. (2016). Co-contribution of hydrogen impurities and native defects might be the answer for the n-type conductivity in ZnO. Phys. Lett. A.

[14] Rana A.U.H.S., Shaikh S.F., Al-Enizic A.M., Agyeman D.A., Ghani F., Nah I.W., Shahid A. (2020). Intrinsic Control in Defects Density for Improved ZnO Nanorod-Based UV Sensor Performance. Nanomaterials.

[15] Ajmal H.M.S., Khan W., Khan F., Huda N.-U., Kim S.-D. (2019). Hydrothermally Grown Copper-Doped ZnO Nanorods on Flexible Substrate. J. Nanoelectron. Optoelectron..

[16] El Khalidi Z., Hartiti B., Siadat M., Comini E., Arachchige H.M.M.M., Fadili S., Thevenin P. (2019). Acetone sensor based on Ni doped ZnO nanostructues: Growth and sensing capability. J. Mater. Sci. Mater. Electron..

[17] Bhati V.S., Hojamberdiev M., Kumar M. (2020). Enhanced sensing performance of ZnO nanostructures-based gas sensors: A review. Energy Rep..

[18] Shabannia R. (2018). High-sensitivity UV photodetector based on oblique and vertical Co-doped ZnO nanorods. Mater. Lett..

[19] Rajalakshmi R., Angappane S. (2013). Synthesis, characterization and photoresponse study of undoped and transition metal (Co, Ni, Mn) doped ZnO thin films. Mater. Sci. Eng. B.

[20] Dai W., Pan X., Chen S., Chen C., Chen W., Zhang H., Ye Z. (2015). ZnO homojunction UV photodetector based on solution-grown Sb-doped p-type ZnO nanorods and pure n-type ZnO nanorods. RSC Adv..

[21] Saleem M., Farooq W.A., Khan M.I., Akhtar M.N., Rehman S.U., Ahmad N., Khalid M., Atif M., Almutairi M.A., Irfan M. (2019). Effect of ZnO Nanoparticles Coating Layers on Top of ZnO Nanowires for Morphological, Optical, and Photovoltaic Properties of Dye-Sensitized Solar Cells. Micromachines.

[22] Archana B., Manjunath K., Nagaraju G., Sekhar K.C., Kottam N. (2017). Enhanced photocatalytic hydrogen generation and photostability of ZnO nanoparticles obtained via green synthesis. Int. J. Hydrogen Energy.

[23] Rahman F. (2019). Zinc oxide light-emitting diodes: A review. Opt. Eng..

[24] Lim J.H., Lee S.M., Kim H.-S., Kim H.Y., Park J., Jung S.-B., Park G.C., Kim J., Joo J. (2017). Synergistic effect of Indium and Gallium co-doping on growth behavior and physical properties of hydrothermally grown ZnO nanorods. Sci. Rep..

[25] Park G.C., Hwang S.M., Lim J.H., Joo J. (2014). Growth behavior and electrical performance of Ga-doped ZnO nanorod/p-Si heterojunction diodes prepared using a hydrothermal method. Nanoscale.

[26] Ali R.N., Naz H., Li J., Zhu X., Liu P., Xiang B. (2018). Band gap engineering of transition metal (Ni/Co) codoped in zinc oxide (ZnO) nanoparticles. J. Alloy. Compd..

[27] Khan W., Khan F., Ajmal H.M.S., Huda N.U., Kim J.H., Kim S.-D. (2018). Evolution of Structural and Optical Properties of ZnO Nanorods Grown on Vacuum Annealed Seed Crystallites. Nanomaterials.

[28] Sengupta J., Sahoo R.K., Bardhan K.K., Mukherjee C. (2011). Influence of annealing temperature on the structural, topographical, and optical properties of sol–gel derived ZnO thin films. Mater. Lett..

[29] Huda N.U., Khan F., Ajmal H.M.S., Khan W., Kim S.-D. (2019). Influence of the N_2_O Plasma Treated ZnO Seed Crystallites on Opto-Electrical Properties of Hydrothermally Grown ZnO Nanorods on Plastic Substrate. J. Nanoelectron. Optoelectron..

[30] Elsayed K.A., Anad N.S., Abdelfattah G., Imam H., Kayed T., Ismail L.Z. (2015). ZnO nanostructures induced by microwave plasma. Arab. J. Chem..

[31] Ajmal H.M.S., Khan F., Huda N.U., Lee S., Nam K., Kim H.Y., Eom T.-H., Kim S.D. (2019). High-Performance Flexible Ultraviolet Photodetectors with Ni/Cu-Codoped ZnO Nanorods Grown on PET Substrates. Nanomaterials.

[32] Ben-Elkamel I., Hamdaoui N., Mezni A., Ajjel R., Beji L. (2018). High responsivity and 1/f noise of an ultraviolet photodetector based on Ni doped ZnO nanoparticles. RSC Adv..

[33] Bairy R., Patil P.S., Maidur S.R. (2019). The role of cobalt doping in tuning the band gap, surface morphology and third-order optical nonlinearities of ZnO nanostructures for NLO device applications. RSC Adv..

[34] Bindu P., Thomas S. (2014). Estimation of lattice strain in ZnO nanoparticles: X-ray peak profile analysis. J. Theor. Appl. Phys..

[35] Theyvaraju D., Muthukumaran S. (2015). Preparation, structural, photoluminescence and magnetic studies of Cu doped ZnO nanoparticles co-doped with Ni by sol–gel method. Phys. E Low-dimensional Syst. Nanostructures.

[36] Ajmal H.M.S., Ramesh S., Kim H.S., Kim S.-D., Choi S.H., Yang W., Kim K.K., Kim H.-S. (2019). Poly(methyl methacrylate)-derived graphene films on different substrates using rapid thermal process: A way to control the film properties through the substrate and polymer layer thickness. J. Mater. Res. Technol..

[37] Yang C.L., Wang J., Ge W.K., Guo L., Yang S., Shen D.Z. (2001). Enhanced ultraviolet emission and optical properties in polyvinyl pyrrolidone surface modified ZnO quantum dots. J. Appl. Phys..

[38] Kegel J., Laffir F., Povey I.M., Pemble M.E. (2017). Defect-promoted photo-electrochemical performance enhancement of orange-luminescent ZnO nanorod-arrays. Phys. Chem. Chem. Phys..

[39] Khan W., Ajmal H.M.S., Khan F., Huda N.U., Kim S.-D. (2018). Induced Photonic Response of ZnO Nanorods Grown on Oxygen Plasma-Treated Seed Crystallites. Nanomaterials.

[40] Rana A.U.H.S., Kim H.-S. (2017). NH_4_OH Treatment for an Optimum Morphological Trade-off to Hydrothermal Ga-Doped n-ZnO/p-Si Heterostructure Characteristics. Materials.

[41] Willander M., Nur O., Sadaf J.R., Qadir M.I., Zaman S., Zainelabdin A., Bano N., Hussain I. (2010). Luminescence from Zinc Oxide Nanostructures and Polymers and their Hybrid Devices. Materials.

[42] Liu W.J., Tang X., Tang Z., Bai W., Tang N.Y. (2013). Oxygen Defects Mediated Magnetism of Ni Doped ZnO. Adv. Condens. Matter Phys..

[43] Djurišić A.B., Leung Y.H., Tam K.H., Ding L., Ge W.K., Chen H.Y., Gwo S. (2006). Green, yellow, and orange defect emission from ZnO nanostructures: Influence of excitation wavelength. Appl. Phys. Lett..

[44] Saravanan R., Santhi K., Sivakumar N., Narayanan V., Stephen A. (2012). Synthesis, and characterization of ZnO and Ni doped ZnO nanorods by thermal decomposition method for spintronics application. Mater. Charact..

[45] Šutka A., Käämbre T., Pärna R., Juhnevica I., Maiorov M., Joost U., Kisand V. (2016). Co doped ZnO nanowires as visible light photocatalysts. Solid State Sci..

[46] Kegel J., Halpin J., Laffir F., Povey I.M., Pemble M.E. (2017). Rapid low-temperature solution growth of ZnO: Co nanorod arrays with controllable visible light absorption. CrystEngComm.

[47] Wang W., Hui S., Zhang F., Wang X., Zhang S., Yan J., Zhang W. (2019). Fabrication and Study on Magnetic-Optical Properties of Ni-Doped ZnO Nanorod Arrays. Micromachines.

[48] Ansari S.A., Ansari S.G., Foaud H., Cho M.H. (2017). Facile and sustainable synthesis of carbon-doped ZnO nanostructures towards the superior visible light photocatalytic performance. New J. Chem..

[49] Park J.H., Lee Y.J., Bae J.-S., Kim B.-S., Cho Y.C., Moriyoshi C., Kuroiwa Y., Lee S., Jeong S.-Y. (2015). Analysis of oxygen vacancy in Co-doped ZnO using the electron density distribution obtained using MEM. Nanoscale Res. Lett..

[50] Zhang Q., Xu M., You B., Zhang Q., Yuan H., Ostrikov K. (2018). (Ken) Oxygen Vacancy-Mediated ZnO Nanoparticle Photocatalyst for Degradation of Methylene Blue. Appl. Sci..

[51] Sarkar S., Basak D. (2013). Defect controlled ultra-high ultraviolet photocurrent gain in Cu-doped ZnO nanorod arrays: De-trapping yield. Appl. Phys. Lett..

[52] Ahmed S. (2017). Structural, optical, and magnetic properties of Mn-doped ZnO samples. Results Phys..

[53] Pimentel A., Ferreira S.H., Nunes D., Calmeiro T., Martins R., Fortunato E. (2016). Microwave Synthesized ZnO Nanorod Arrays for UV Sensors: A Seed Layer Annealing Temperature Study. Materials.

[54] Shabannia R. (2016). Synthesis and characterization of Cu-doped ZnO nanorods chemically grown on flexible substrate. J. Mol. Struct..

[55] Alenizi M.R., Henley S.J., Silva S. (2015). On-chip Fabrication of High Performance Nanostructured ZnO UV Detectors. Sci. Rep..

[56] West C., Robbins D., Dean P., Hayes W. (1983). The luminescence of copper in zinc oxide. Phys. B+C.

[57] Chang S., Chang M., Yang Y. (2017). Enhanced responsivity of GaN metal-semiconductor-metal (MSM) photodetectors on GaN substrate. IEEE Photon J..

[58] Dong Y., Zou Y., Song J., Li J., Han B., Shan Q., Xu L., Xue J., Zeng H. (2017). An all-inkjet-printed flexible UV photodetector. Nanoscale.

[59] Lai W.-C., Chen J.-T., Yang Y.-Y. (2013). Optoelectrical and low-frequency noise characteristics of flexible ZnO-SiO2 photodetectors with organosilicon buffer layer. Opt. Express.

[60] Ji C.H., Kim K.T., Oh S.Y. (2018). High-detectivity perovskite-based photodetector using a Zr-doped TiO: X cathode interlayer. RSC Adv..

[61] Sugumar R., Angappane S. (2017). Influence of substrate heating and annealing on the properties and photoresponse of manganese doped zinc oxide thin films. Superlattices Microstruct..

